# Fetal nuchal translucency: is there an association with birthweight and neonatal wellbeing?

**DOI:** 10.4274/tjod.galenos.2019.21384

**Published:** 2019-03-27

**Authors:** Ziya Kalem, Aşkı Ellibeş Kaya, Batuhan Bakırarar, Müberra Namlı Kalem

**Affiliations:** 1 Gürgan Clinic IVF and Women Health Center, Ankara, Turkey; 2 Düzce University Faculty of Medicine, Department of Obstetrics and Gynecology, Düzce, Turkey; 3 Ankara University, Department of Biostatistics, Ankara, Turkey; 4 Bahcesehir University, Department of Obstetrics and Gynecology, İstanbul, Turkey

**Keywords:** Nuchal translucency, birthweight, APGAR score, fetal development

## Abstract

**Objective:**

To evaluate the relationship between nuchal translucency (NT) values with birthweight and the wellbeing of the newborn.

**Materials and Methods:**

This retrospective cohort study that included 508 patients made use of data on healthy full-term, singleton, live birth newborns in a university hospital between 2016 and 2018. The relationship between the NT multiple of the median (MoM) value and maternal body mass index, birthweight, sex, need for neonatal intensive care unit (NICU), and APGAR scores was evaluated. Similarly, the relationship between birthweight and NT MoM, and biochemical data in the first trimester was also evaluated.

**Results:**

There was a positive correlation between NT and birthweight (p<0.001). The need for NICU admission increased (p=0.001), and APGAR 1st minute scores decreased (p=0.001) with increasing NT, and APGAR 5th minute scores remained unchanged (p=0.057).

**Conclusion:**

The present study identified a positive correlation between first trimester NT and birthweight, and a negative correlation with the wellbeing of the neonate.

**PRECIS:** Nuchal translucency: association with birthweight and neonatal wellbeing?

## Introduction

Nuchal translucency (NT) is the normal fluid-filled subcutaneous space identified at the back of the fetal neck during the late first trimester and early second trimester^(1)^. NT is one of the parameters of first trimester screening tests for trisomies 21, 18 and 13^(2)^, and an increased NT is thought to be related to dilated lymphatic channels and is considered a nonspecific sign of a more generalized fetal abnormality^(3)^. Fetuses with an increased NT are at increased risk of chromosomal anomalies, structural defects, and genetic syndromes^(4,5)^.

Recent studies have reported that despite increased NT in the first trimester in fetuses with normal karyotypes, no problem will be experienced in the long term if ultrasonographic (USG) scans show normal findings in the second trimester; however, there is an increased probability of adverse pregnancy outcomes^(6,7)^. Although noninvasive prenatal diagnosis tests have largely replaced first-trimester screening tests, the measurement of NT in the first trimester will always maintain importance. A future protocol is considered to involve USG screening in the second trimester, directed by the noninvasive prenatal tests and NT measurements in the first trimester^(8)^.

Although there are ongoing studies in literature attempting to determine cut-off values for NT in healthy fetuses with no chromosomal anomalies, researchers have also noticed that NT values vary depending on race and fetal development^(9,10,11,12)^. It is now thought that differences in fetal body measurements, even NT, can be observed from the first trimester onwards^(13)^.

The present study investigated whether NT values measured in the first trimester varied such as other measurements regarding the body or if it indicated adverse pregnancy events. To this end, the study investigated the relationship between NT values with birthweight and the wellbeing of the newborn.

## Materials and Methods

This retrospective cohort study made use of data on healthy full-term, singleton, live birth newborns in a university hospital between 2016 and 2018. The study included 508 patients for whom postnatal and first trimester screening test findings were available. Newborns with congenital anomalies or systemic diseases diagnosed in the intrauterine or postpartum period, and those underwent karyotype analysis for any reason during pregnancy or postpartum and who were found to have an abnormal karyotype were excluded from the study. Patients with indefinite last menstrual periods and those with USG measurements that were inconsistent with the last menstrual period, *in vitro *fertilization (IVF) patients, patients who received exogenous progesterone in the first trimester, multiple pregnancies, patients delivering their babies before 37 weeks of gestation, newborns with known intrauterine viral infections, patients with preeclampsia/eclampsia, and diabetic mothers were also excluded from the study. The maternal age, height, weight, data on previous pregnancies, crown-rump-length measurements (mm), pregnancy-associated plasma protein A multiples of median (PAPP-A MoM) values, free beta human chorionic gonadotropin (hCG) MoM values, and NT MoM values were used for abnormal karyotype screening tests in the first trimester, maternal weight gain, newborn’s APGAR scores, need for neonatal intensive care unit (NICU), sex, and birthweight of those involved in the study were recorded. The relationship between the NT MoM values and maternal body mass index (BMI), birthweight, sex, and APGAR scores was evaluated. Similarly, the relationship between birthweight and NT MoM and biochemical data in the first trimester was also evaluated.

### Statistical Analysis

All statistical analyses were performed using SPSS for Windows 11.5 software program (SPSS Inc., Chicago, IL). Compatibility of data with normal distribution was examined graphically and using the Kolmogorov-Smirnov test. Mean ± standard deviation and median (min-max) were used for the quantitative variables, an numbers (percentage) were used as descriptors for categorical variables in the study. When to look whether there was a statistically significant difference between the categories of a qualitative variable with two categories in terms of a quantitative variable, Student’s t-test was used if the normal distribution assumption was met; if not, Mann-Whitney U test was used. The chi-square test was used to examine the relationship between two categorical variables. Covariance analysis (ANCOVA) was used to see whether one or more continuous independent parameters had any impact on the dependent parameter. Receiver operating charcteristics curve analysis was used to find the discriminative factors between the groups. Significance level was set at p=0.05.

## Results

The study included 508 subjects, the demographic characteristics of whom and the parameters evaluated in the study are presented in Table 1.

Of these subjects, 25 (4.9%) were current smokers. The nasal bone could not be visualized in 22 subjects (4.3%). Of the newborns, 267 (52.5%) were male and 241 (47.4%) were female. Of the 21 (4.1%) babies that were required to be admitted to the NICU, 15 (71.4%) had respiratory problems and five (23.8%) had other system problems (gastrointestinal, cardiovascular); four babies (0.7%) died in the neonatal period.

Multiple linear regression analysis was performed to evaluate the relationship between NT MoM values and maternal BMI, and the parameters of the newborn (Table 2). In the multiple linear regression model: NT MoM: 0.687+0.001 * newborn’s weight + 0.503 * need for NICU + (-0.025) * newborn’s APGAR 1 min, and the p values for the variables were <0.001, <0.001, and 0.015, respectively. When the variables were incorporated into the model together, they accounted for 15.6% of the variance in the NT MoM variable.

Linear regression analysis was made to evaluate the relationship between newborn weight with maternal parameters and NT MoM (Table 3). In the multiple linear regression model: newborn weight: 2896.277 + 16.031 * weight gain + (-331.941) * smoking + 248.250 * Nt MoM, and the p values for the variables were 0.003, 0.001, and <0.001, respectively. When these variables were incorporated into the model together, they accounted for 9.2% of the variance in the newborn weight.

The variables were added individually to the model in a logistic regression analysis performed to evaluate the effects of maternal and fetal factors on the need for admission to the NICU (Table 4). The logistic regression model was statistically significant (p<0.001), and explained 30.4% (Nagelkerke *R2*) of the variance in the need for NICU, and accurately classified 96.7% of patients. Increasing maternal age and NT MoM were associated with an increase in the likelihood of requiring admission to the NICU.

## Discussion

The present study evaluates the relationship between NT in the first trimester and newborn weight, and found a significant positive correlation between the two. The study also evaluates the relationship between NT in the first trimester and the wellbeing of the newborn, and found that an increase in NT was associated with an increase in the need for admission to the NICU and a decrease in the APGAR 1^st^ minute score.

It is currently acknowledged in the literature that variations in fetal weight occur from the first trimester onwards, rather than the traditionally accepted second half of the second trimester, fetal measurements other than NT have been used in these studies^(13,14,15,16)^. Studies in the literature have linked increased first trimester NT to adverse pregnancy outcomes after being linked initially to fetal birthweight over complications accruing during pregnancy. Krantz et al.^(17)^ showed that PAPP-A and increased NT could be associated with adverse pregnancy outcomes, and particularly with intrauterine growth retardation (IUGR)^(17)^, and another study suggested that increased NT together with impaired glucose intolerance could reflect fetal macrosomia^(18)^.

In later years, studies began to report an association between NT and birthweight in the absence of adverse pregnancy outcomes. In a study of the non-diabetic population, it was shown that an increased first trimester NT was linked to large-for-gestational-age (LGA) neonates^(19)^. In a study published in 2017 by Hackmon et al.^(20)^, it was reported that early fetal measurements and NT were correlated with term birthweight^(20)^. Given that previous studies faced problems in accurately determining gestational age, this study involved IVF patients so as to ensure the accuracy of gestational age.

The present study established a positive association between first trimester NT and birthweight, although our study includes non-IVF patients. Some studies in literature demonstrated increased NT with the use of exogenous progesterone in the first trimester^(21,22)^, and it is widely known that progesterone is used in the luteal phase and the first trimester as a worldwide standard procedure in IVF patients^(23,24)^. Accordingly, the current study population was selected from non-IVF patients and those who did not use progesterone in the first trimester. For an accurate determination of gestational age, patients with a last menstrual period that was consistent with USG measurements were selected.

Plasencia et al.^(25)^ reported an association between birthweight and NT, serum PAPP-A, beta hCG, and uterine artery Doppler PI measurements, and suggested that these findings could lead to the early recognition of LGA infants. The present study did not include Doppler USG findings; however, we identified no association between parameters other than NT (PAPP-A and beta hCG) and birthweight.

The findings of the present study identified no relationship between NT and fetal sex, although there have been studies in literature in which such a relationship was demonstrated. Spencer et al. reported that NT was 3.4% lower in female fetuses^(26)^, whereas Weismann-Brenner et al.^ (27)^, in contrast, found that NT was higher in the male sex, and that the correlation between NT and birthweight was independent of sex. Such a relationship could not be reliably established in the present study due to the small number of participants.

Aside from its relationship with NT, the present study identified a positive correlation between birthweight and maternal weight gain, and a negative correlation with smoking, and both of these findings are consistent with literature^(28,29,30)^.

In the present study, it was observed that the need for NICU admission increased and APGAR 1^st^ minute scores decreased with increasing NT, but APGAR 5^th^ minute scores remained unchanged. Previous studies suggest that if U scans in the second trimester and afterwards are normal in babies with increased first trimester NT and normal karyotypes, there will be no adverse outcome in the long term, although there is an increased likelihood of adverse pregnancy outcomes^(6,7,31)^. Studies have also shown that the long-term neurodevelopmental outcome of children after increased fetal NT is reassuring^(32)^.

The APGAR 5-minute score is also a good indicator of a long-term neurodevelopmental outcome^(33)^, and its lack of variance to NT in the present study is an expected finding. The APGAR 1^st^ minute score determines how well the baby tolerated the birthing process^(34)^, and the relationship between increased NT and the APGAR 1^st^ minute score identified in the present study suggests that babies with increased NT may have poorly tolerated the birthing process. This indicates that a relationship may exist between increased NT and placental insufficiency. In a recently published study, Lee et al.^ (35)^ evaluated the relationship between NT and placental dysfunction and reported higher first trimester NT in babies exhibiting signs of placental insufficiency, although this was not statistically demonstrated. It would seem that this topic deserves further comprehensive research.

The increased need for NICU admission with increasing NT values in the present study indicates that placental insufficiency develops and tolerance to the birthing process decreases with increasing NT MoM values. Our study has demonstrated two factors that affect the need for NICU admission, namely increasing maternal age and increasing NT. It is, however, unknown whether a relationship exists between increasing maternal age and increasing NT in babies with normal karyotypes. The increasing rate of miscarriage with increasing maternal age complicates a joint evaluation of these two factors, but it is clear that this area merits more comprehensive research.

Studies in the literature have reported an increased likelihood of submicroscopic chromosomal abnormalities in fetuses with increased NT and normal karyotypes, and that this could cause structural defects in the second and third trimesters. The placenta may be affected by these microscopic chromosomal abnormalities, and accordingly, may cause adverse pregnancy outcomes such as preeclampsia and IUGR^(36)^.

The present study is the first in the literature to investigate the relationship between the first trimester NT MoM values of healthy newborns with both the birthweight and the wellbeing of the newborns. The most important limitation of the current study is that no karyotype analysis was available for all of the newborns in the study. Furthermore, no long-term data related to the newborns in the study were included, other than the neonatal period. The retrospective nature, the lack of details for the indications of NICU admission, and lack of a retrospective power analysis also limits of this study.

In conclusion, the present study identified a positive correlation between first trimester NT and birthweight, and a negative correlation with the wellbeing of the neonate. A number of studies in literature have aimed at determining cut-off levels for NT; these studies should take into account the variations in NT values. It is not yet understood whether different NT values indicate a variation in fetal development or an adverse perinatal outcome, and so further studies are required to clarify this issue.

## Figures and Tables

**Table 1:**
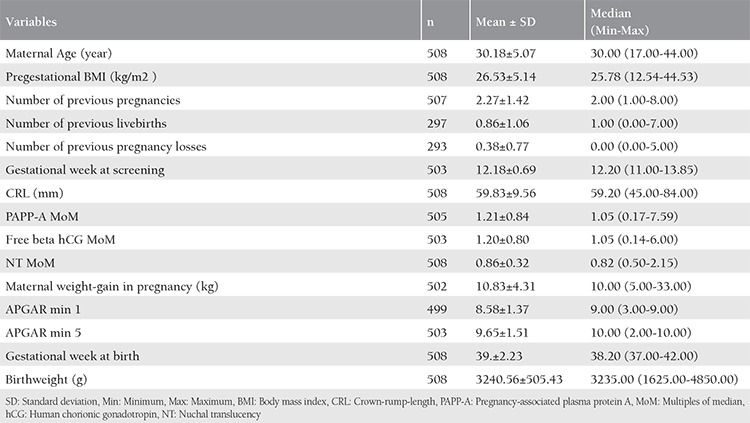
Demographic data, parameters related to the pregnancy and the newborn

**Table 2:**
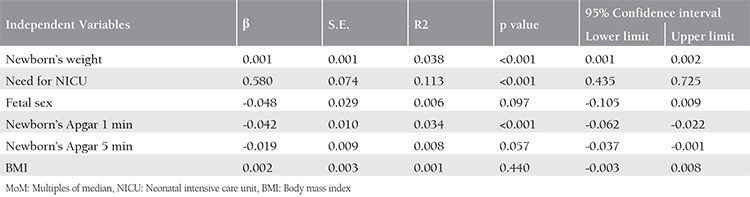
Nuchal translucency MoM-when the variables are included in the model one at a time

**Table 3:**
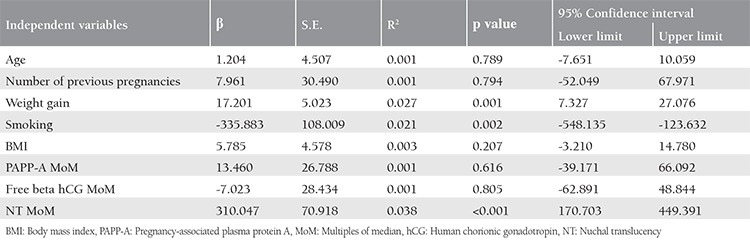
Newborn’s Weight-when the variables were included in the model one at a time

**Table 4:**
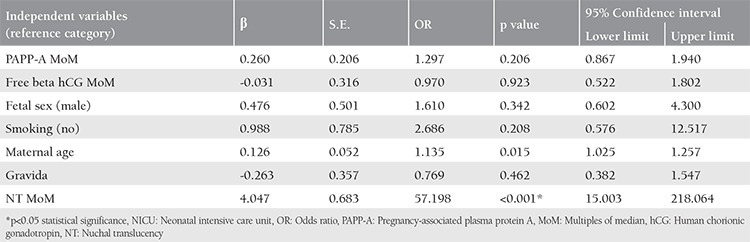
Need for NICU-When the variables are included in the model one at a time
